# Impact Evaluation of DME Beacons on BeiDou B2a Signal Reception Performance

**DOI:** 10.3390/s25123763

**Published:** 2025-06-16

**Authors:** Yicheng Li, Jinli Cui, Zhenyang Ma, Zhaobin Duan

**Affiliations:** 1 Key Laboratory of Civil Aviation Aircraft Airworthiness Certification Technology, Civil Aviation University of China, Tianjin 300300, China; ycli@cauc.edu.cn (Y.L.); zbduan@cauc.edu.cn (Z.D.); 2 College of Safety Science and Engineering, Civil Aviation University of China, Tianjin 300300, China; 2023091084@cauc.edu.cn; 3 Institute of Science and Technology Innovation, Civil Aviation University of China, Tianjin 300300, China

**Keywords:** B2a, carrier-to-noise ratio (C/N0), DME, digital pulse blanker, pulse interference, signal reception performance

## Abstract

The operational integrity of the BeiDou-3 Navigation Satellite System (BDS-3) has been significantly challenged by electromagnetic interference, particularly from Distance Measuring Equipment (DME) ground beacons to the newly implemented B2a signal, since its full operational deployment in 2020. This study developed a comprehensive interference evaluation model based on receiver signal processing principles to quantify the degradation of B2a signal reception performance under DME interference scenarios. Leveraging empirical data from the DME beacon network in the Chinese mainland, we systematically analyzed the interference effects through an effective carrier-to-noise ratio ($C/N_{0}$), signal detection probability, carrier tracking accuracy, and demodulation bit error rate (BER). The results demonstrate that the effective $C/N_{0}$ of the B2a signal degrades by up to 3.25 dB, the detection probability decreases by 33%, and the carrier tracking errors and BER increase by 2.57° and 5.1%, respectively, in worst-case interference scenarios. Furthermore, significant spatial correlation was observed between the interference hotspots and regions of high aircraft density. DME interference adversely affected the accuracy, availability, continuity, and integrity of the airborne BeiDou navigation system, thereby compromising civil aviation flight safety. These findings establish a scientific foundation for developing Minimum Operational Performance Standards for B2a signal receivers and for strategically optimizing DME beacon deployment throughout the Chinese mainland.

## 1. Introduction

**Figure 1:**
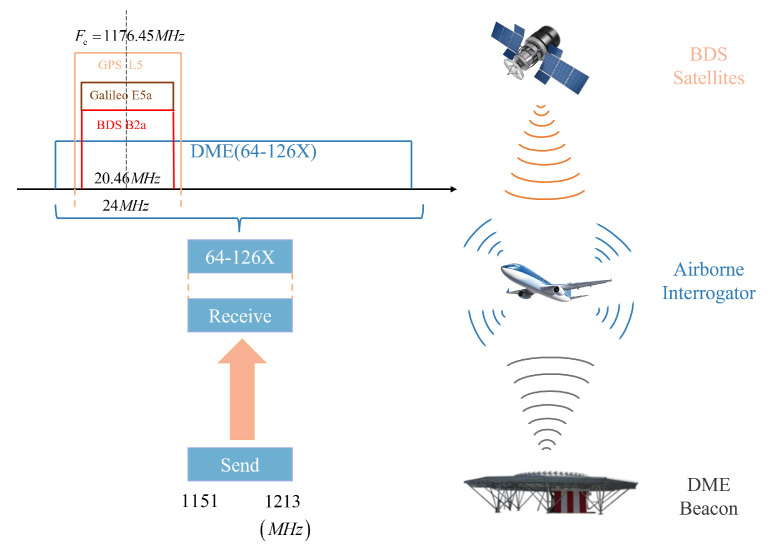
The spectrum conflict between DME and GNSS signals.

Global Navigation Satellite Systems (GNSS) signal processing is susceptible to electromagnetic interference, which can severely degrade signal quality, positioning accuracy, and overall system reliability [1,2,3]. In the context of civil aviation, identifying and quantifying the impact of radio frequency interference (RFI) on the performance of navigation signals reception is essential [4,5]. The BeiDou-3 Navigation Satellite System (BDS-3), which achieved full operational capability in 2020, marked a major milestone in China’s satellite navigation technology. Operating at 1176.45 MHz, the newly implemented B2a signal is spectrally compatible with Global Positioning System (GPS) L5 and Galileo E5a signals through identical frequency allocation and similar modulation characteristics. This enables improved positioning accuracy and precise timing for airborne navigation systems [6]. However, the frequency resources available for aeronautical radio navigation services are highly constrained and exhibit significant overlap with the operating frequency bands of ground-based navigation systems. Distance Measuring Equipment (DME), serving as essential ground-based navigation infrastructure, operates in the 1151–1213 MHz frequency band. This range creates substantial spectral overlap with the B2a signal (Figure 1), potentially causing significant interference. Consequently, DME has emerged as a primary RFI source for B2a signal in aviation applications [7].

Previous studies have confirmed the existence of RFI between DME and GNSS signals (GPS L5/BeiDou B2 bands) [8,9]. It has been shown that the level of interference caused by DME beacons to aeronautical users is closely related to the geographical distribution of DME ground beacons [10,11]. In high-altitude flight scenarios, aeronautical users are subject to the greater interference that results from the simultaneous reception of multiple DME ground beacon signals [12]. To quantify the interference effects of DME under worst-case scenarios, the Radio Technical Commission for Aeronautics (RTCA) evaluated the degradation of the carrier-to-noise ratio ($C/N_{0}$) of GNSS L5/E5a signals in high-altitude flight conditions [13]. The International Civil Aviation Organization (ICAO) conducted further studies on DME beacons in Europe and the United States, which clarified the impact distribution of European and US DME ground beacons on L5/E5a signal performance at high altitudes [14,15].

The global deployment of BDS-3 has driven intensified research into B2a signal interference characteristics, particularly in aeronautical navigation contexts [16,17,18]. Wang et al. proposed a method to evaluate B2a signal $C/N_{0}$ degradation. Their approach specifically analyzed the interference from DME beacons in potential interference hotspots of the Chinese mainland, with particular consideration given to complex terrain effects [16]. Liu et al. conducted a systematic analysis of DME-induced $C/N_{0}$ degradation mechanisms in airborne receivers across different parameter configurations, extending the investigation to its consequential effects on aviation protection level performance [17]. Zhang et al. conducted a quantitative analysis of B2a signal interference from DME beacons co-located with VORs in the Chinese mainland based on the RTCA assessment framework [18]. However, this model is limited to single-parameter ($C/N_{0}$) evaluation and exhibits conservative estimations under multi-DME interference scenarios [19]. While significant progress has been made in interference assessment approaches and impact quantification, several critical issues remain to be addressed. The current evaluation systems primarily focus on a single dimension of $C/N_{0}$ degradation, lacking systematic consideration of the impacts that are present throughout the entire signal processing chain. The spatial scope was limited to preliminary analyses of specific hotspots or localized regions, with no comprehensive quantitative assessment of the DME beacons across the entire Chinese mainland, and it was also found that signal propagation loss factors need to be more thoroughly incorporated into existing models. The research related to investigating the DME effects on B2a, compared with GPS and Galileo, is still relatively limited. The general aim of this paper was, thus, to develop an enhanced evaluation model for quantifying the impact of DME beacons on B2a signal reception performance across the whole area of the Chinese mainland. Quantitative analysis of the multi-parameter performance degradation for airborne users caused by multiple DME interference in high-altitude scenarios was conducted with determination of the worst-case interference values.

In this study, a mathematical model was first constructed based on the principle of pulse blanker to quantify the B2a signal $C/N_{0}$ degradation under DME interference scenarios. Taking into account the signal processing process of BeiDou receivers, an evaluation model was established to assess the degradation of B2a signal reception performance. Subsequently, the degradation of B2a signal reception performance and the distribution of interference hotspots were evaluated using empirical data from the DME beacon network in the Chinese mainland and the interference budget model. The findings establish a scientific foundation for developing Minimum Operational Performance Standards for B2a signal receivers and strategically optimizing DME beacon deployment across the Chinese mainland.

## 2. DME Interference Model

DME operates across the 962–1213 MHz frequency spectrum, utilizing 252 discrete channels with 1 MHz inter-channel spacing. The system employs two distinct operational modes: X-mode and Y-mode. For airborne equipment, the transmission frequency for channels 1X to 126X ranges from 1025 MHz to 1150 MHz, while the reception frequencies range from 962 MHz to 1024 MHz (for channels 1X–63X) and from 1151 MHz to 1213 MHz (for channels 64X–126X) [20]. The B2a signal completely falls within 1151 MHz to 1213 MHz, and the DME interference is mainly from ground beacons [13]. Consequently, this study specifically examined the interference from DME ground beacons operating in channels 64X–126X (1154–1213 MHz).

**Figure 2:**
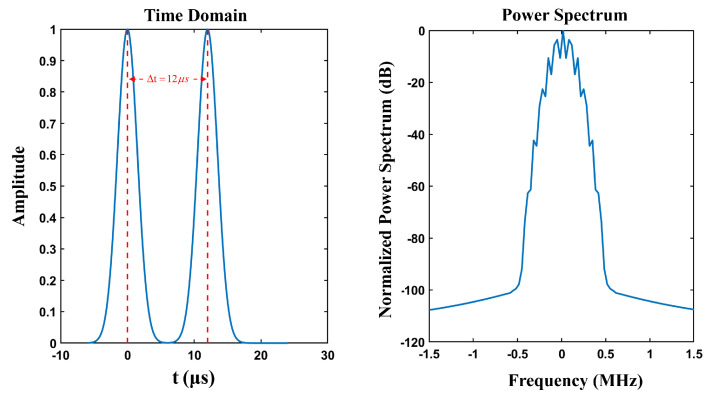
The time domain waveform and spectral characteristics of the DME pulse pairs.

The DME signal consists of repetitive pulse pairs, and the time interval between the X-mode pulse pairs is 12 μs. The signal expression for the DME X-mode can be expressed by

(1) $$S_{DME}\left(t\right)=e^{\frac{\alpha t^{2}}{2}}+e^{-\frac{\alpha {\left(t-\Delta t\right)}^{2}}{2}},$$

where α is $4.5\times 10^{11}\;s^{-2}$, indicating the width of the pulse; and Δt=12 μs is the time interval between two pulses. The time-domain waveform and spectral characteristics of the simulated signal are shown in Figure 2. Pulse pairs arrived randomly at the aircraft, and the output peak power was 1 kW.

To describe the pulse pairs’ transmission rate of the ground beacon, the number of pulse pairs transmitted by a ground beacon per second, i.e., the pulse repeat rate (PRR), was usually used. As aircrafts may experience interference from different ground beacons at the same time, we designate the beacons as 1, 2, …, I. The DME radio frequency signal as interference can be defined as follows [15]:

(2) $$j\left(t\right)=\sum_{i=1}^{I}\sqrt{P_{r}^{i}}S_{p}^{i}\left(t\right)\cos(2\pi f_{i}t+\phi_{i},$$

where $S_{p}^{i}\left(t\right)$ denotes the complete baseband signal transmitted by the i-th DME beacon; and $P_{r}^{i}$, $f_{i}$, and $\phi_{0}$ denote the peak power, carrier frequency, and carrier phase of the i-th DME signal, respectively.

If only the influence of DME interference is considered, the signal r(t) received by the BDS receiver antenna can be expressed by

(3) r(t)=s(t)+j(t)+n(t,

where s(t) denotes the BeiDou signal, and n(t) denotes white noise.

## 3. Interference Evaluation Model

While the aforementioned DME interference model characterized the interference source properties, its actual impact on B2a signal reception performance required further quantification through the receiver processing chain. This section analyzes how DME pulses affect the carrier-to-noise ratio and reception performance of the B2a signal through critical modules, including the pulse blanker. The derivation of the interference evaluation model was finally completed.

The reception performance of the GNSS signals primarily encompasses acquisition, tracking, and demodulation, which mainly depends on the signal-to-noise-plus-interference-ratio (SNIR) at the output of the instant correlator in the receiver. However, due to the complexity of analyzing the SNIR, the $C/N_{0}$ was frequently utilized to characterize the signal [21,22]. The degradation in signal reception performance can be further calculated once the $C/N_{0}$ degradation is known.

### 3.1. Generic Airborne Civil Aviation BDS Receiver

**Figure 3:**
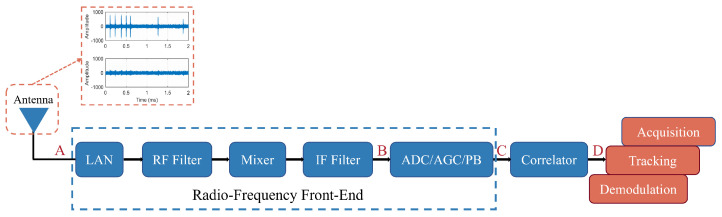
The structure of a general civil aviation BDS receiver.

To support the evaluation model, this paper described a generic structure of the airborne civil aviation BDS receiver (as shown in Figure 3) and analyzed the impact of its key components on the received signal.

The composite received signals were captured at the antenna port (Point A) and processed by the radio frequency front-end (RFFE) block (Point B). Within the RFFE, the signal underwent low-noise amplification, and this was followed by frequency downconversion to an intermediate frequency (IF) and multi-stage filtering for image rejection and spurious signal suppression. A digital pulse blanker (PB) was introduced after the RFFE block (Point C), which is key for the pulse interference suppression in the receiver [23]. The ideal PB operates as a zero-latency comparator that suppresses the signal to zero when the input power exceeds a predefined threshold. The blanking operation nullifies all input components, including useful signals, interference, and noise. While effectively reducing noise power, it also diminishes the peak amplitudes of useful signals. Consequently, the overall $C/N_{0}$ experiences degradation [24]. The digitized and blanked signals are then fed to the correlator. At this stage, the residual RFI components present at the correlator output (Point D) will degrade the navigation signal reception performance.

### 3.2. Effective Carrier-to-Noise Ratio Degradation

Considering the impact of receiver components on the signals, the $C/N_{0}$ was defined as the ratio of the carrier signal power to the noise power at the correlator output of the receiver’s carrier tracking loop [25,26]. The degradation of the effective carrier-to-noise ratio, denoted as ${\left(C/N_{0}\right)}_{\deg}$, was defined as the difference between $C/N_{01}$, which only represents the carrier-to-noise ratio when the desired signal is present at the receiver antenna port, and $C/N_{02}$, which corresponds to the carrier-to-noise ratio when a mixture of the desired signal and RFI is present (with pulse blanking enabled). This parameter is crucial for characterizing the impact of interference on navigation signals. The ${\left(C/N_{0}\right)}_{\deg}$ caused by DME interference is derived as [13]

(4) $${\left(C/N_{0}\right)}_{\deg}=-10\log_{10}\left(\frac{1-PDC}{1+\frac{I_{0,\mathrm{WB}}}{N_{0}}+Ri}\right),$$

where $I_{0,WB}$ represents the total continuous aeronautical RFI power spectral density (PSD), and $N_{0}$ is the thermal noise power spectral density of the GNSS receiver. The key parameters governing DME interference are the pulse blanker duty cycle (PDC) and the interference-to-noise power spectral density ratio below the blanking threshold (Ri), both of which are determined by the receiver’s pulse blanker characteristics. Assuming that the received peak power of the DME pulse signal is $P_{k}$ and that it is converted into an equivalent rectangular pulse, with the PB threshold set to $P_{th}$, the modeling of PDC and Ri is as follows.

#### 3.2.1. Single DME Signal Interference

**Figure 4:**
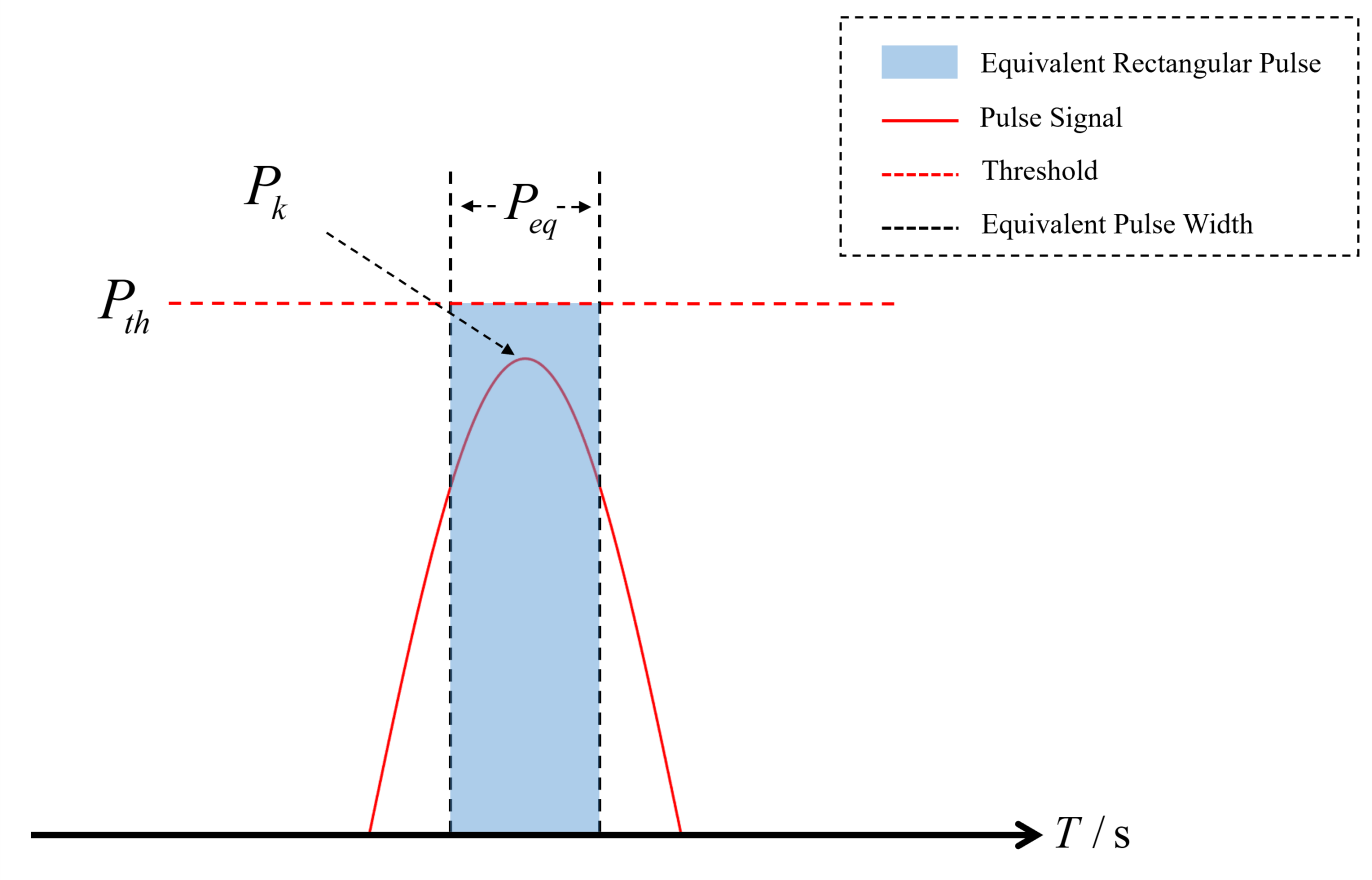
Blanking of a weak pulse signal.

For the case where $P_{k}\leq P_{th}$, as shown in Figure 4, we have the following:

When the received signal power remains below the blanking threshold, the PB maintains inactive status, resulting in $PDC_{i}=0$. The width of the equivalent rectangular pulse can be obtained by directly integrating the signal:

(5) $$P_{eq}=\int_{-\infty }^{\infty }e^{-\alpha t^{2}}dt.$$

The equivalent rectangular pulse width of a single pulse is 2.6 μs.

The DME pulse transmits 2700 pulse pairs per second ($\lambda_{\mathrm{PRR}}=2700$), and the equivalent pulse signal power per second is

(6) $$dc_{i}=2700\times 2\times P_{eq}.$$

According to the RTCA calculations, the thermal noise power spectral density of the GNSS receiver $N_{0}$ was −200 dBW [13]. For the B2a signal receiver, the bandwidth BW was 20 MHz [6], where $R_{i}$ can be expressed as

(7) $$Ri_{i}=\frac{P_{k}\times dc_{i}}{N_{0}\times BW}.$$

**Figure 5:**
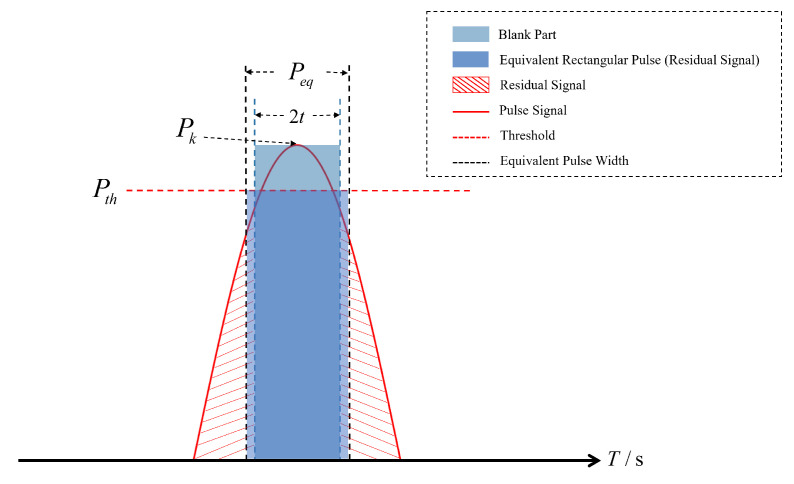
Blanking of a strong pulse signal.

For another case where $P_{k}\geq P_{th}$, as shown in Figure 5, we have the following:

It must be noted that $dc_{i}$ and $R_{i}$ follow Formulas (6) and (7). Since the PB was triggered, the equivalent rectangular pulse width could not be determined through integration. For the pulse width of the blanking part, the power of a single pulse pair was made equal to the threshold:

(8) $$P_{k}e^{-\alpha t^{2}}=P_{th}.$$

It can be calculated that

(9) $$t=\pm \sqrt{\frac{\ln(P_{k}/P_{th})}{\alpha }}.$$

After pulse blanking, the signal only retains residual components beyond the eliminated main lobe, as shown in the red shaded part in Figure 5. The area of the equivalent rectangular pulse in the figure is equal to the area of the signal residual part. The equivalent rectangular pulse width of the residual signal can be obtained from the complementary error function (ERCF):

(10) $$P_{eq}=\frac{\mathrm{erfc}(w\sqrt{\alpha })}{\sqrt{\alpha }},$$

where *w* is half of the pulse blanking width, which is equal to *t* in value. Subsequently, the equivalent rectangular pulse width was used to calculate the value of PDC:

(11) $$PDC_{i}=P_{eq}\times \lambda_{PRR}.$$

**Figure 6:**
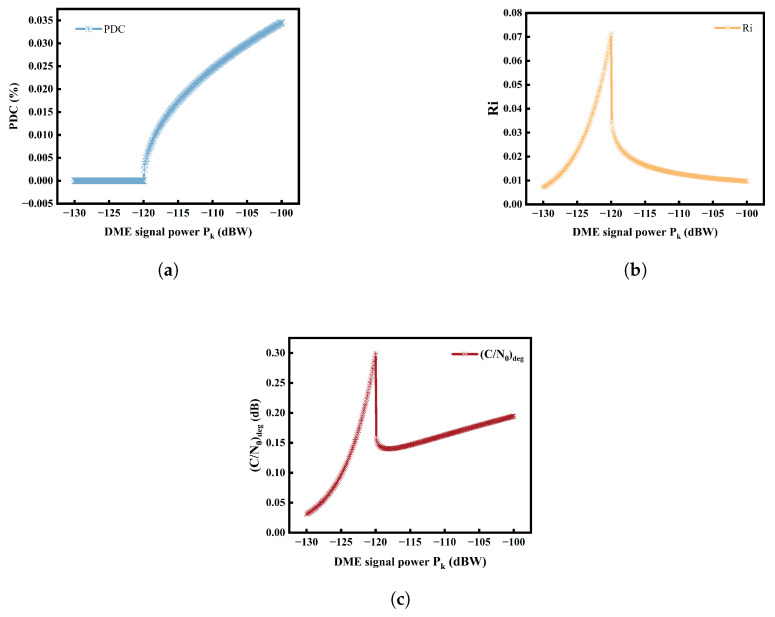
The impact of the DME signal received power on PDC (**a**), $R_{i}$ (**b**), and ${\left({C/N}_{0}\right)}_{\deg}$ (**c**).

The performance of the PB was directly related to the selection of the pulse blanking threshold. According to RTCA validation, the receiver achieved better overall performance when $P_{th}=-120$ dBW [13], and this value has been implemented in practical receivers. Additionally, the calculated total continuous aviation RFI power spectral density was $I_{0,WB}=-197.5$ dBW/Hz [13]. By incorporating these parameters, the ${\left(C/N_{0}\right)}_{\deg}$ values under different interference power levels for a single DME signal were obtained, as illustrated in Figure 6.

The PDC remained a zero value before the PB was triggered. However, both $R_{i}$ and ${\left({C/N}_{0}\right)}_{\deg}$ exhibited increases progressively as the interfering signal persisted and $P_{k}$ gradually increased. $R_{i}$ and ${\left({C/N}_{0}\right)}_{\deg}$ reached the maximum value when $P_{k}$ equaled $P_{th}$. When $P_{k}>P_{\mathrm{th}}$, PDC began to show positive values due to the blanking effect of PB, while $R_{i}$ and ${\left({C/N}_{0}\right)}_{\deg}$ decreased as the interference signals diminished. However, when $P_{k}$ became excessively large, the overactive blanking effect led to a significant reduction in the B2a signal power, while the ${\left({C/N}_{0}\right)}_{\deg}$ gradually resumed an upward trend.

#### 3.2.2. Multiple DME Signal Interference

Aviation users can receive signals from different DME beacons at a given position in real flight scenarios. Assuming that the arrival times of different DME pulse signals at the receiver are uniformly distributed and do not interact, the total PDC and Ri can be calculated as

(12) $$PDC=1-\prod (1-PDC_{i}),$$

(13) $$Ri=\sum_{i}Ri_{i},$$

where *i* denotes the number of DME interference signals received by the airborne receiver.

### 3.3. B2a Signal Reception Performance Degradation

Detection probability is a key parameter of signal acquisition and determines the acquisition performance [27]. If $H_{0}$ represents noise only and $H_{1}$ represents a composite signal consisting of both interference and noise, the power of the input signal, which is defined as the detection metric, can be expressed as

(14) $$z=\sum_{j=1}^{M}\left(I_{j}^{2}+Q_{j}^{2}\right).$$

The intermediate frequency (IF) signal received by the receiver is mixed with the in-phase and quadrature carriers, respectively, to obtain the I-channel and Q-channel signals. $I_{i}$ and $Q_{i}$ are the coherent integration results for the *I* and *Q* paths, respectively, while *M* is the number of incoherent integrations.

The detection threshold was $V_{t}$, and the false alarm probability and detection probability of the signal can be expressed by

(15) $$P_{f}=\int_{V_{t}}^{\infty }\frac{1}{2^{M}\Gamma \left(M\right)}z^{M-1}\exp\left(-\frac{z}{2}\right)dz,$$

(16) $$P_{d}=\int_{V_{t}}^{\infty }\frac{1}{2}{\left(\frac{z}{\lambda }\right)}^{\frac{M-1}{2}}\exp\left(-\frac{z+\lambda }{2}\right)I_{M-1}\left(\sqrt{z\lambda }\right)dz,$$

where Γ(M)=(M−1)! is the gamma function; $\lambda =2M\left(S/N_{0}\right)=2M\left(C/N_{0}\right)T_{coh1}$ is the noncentral parameter of the noncentral chi-squared distribution; $T_{coh1}$ represents the pre-detection integration time; and $I_{M-1}=\left(\sqrt{z\lambda }\right)$ is the first class of M−1 order Bessel functions.

In the practical acquisition process, the false alarm probability $P_{f}$ for signal detection should be minimized; typically, it is set to $P_{f}=1\times 10^{-5}$. And the detection probability of the signal under different detection integration times can be further calculated if $P_{f}$ and $C/N_{0}$ are known.

The acquired navigation signal iswas passed into the carrier tracking loop, where its performance governed both tracking precision and dynamic response. The most commonly used carrier tracking ring was a phase locked loop (PLL), and its tracking quality was quantified by the carrier tracking measurement error [28]. The jitter sources of the PLL are generally transient for generic airborne civil aviation BDS receivers, and carrier tracking errors are typically attributed to thermal noise. When interference exists, its contribution to carrier tracking error must be additionally accounted for. The final carrier tracking error caused by thermal noise and interference can be determined by

(17) $$\delta_{PLL}=\frac{360}{2\pi }\sqrt{\frac{B_{P}}{C/N_{0}}\left(1+\frac{1}{2T_{\mathrm{coh}2}C/N_{0}}\right)},$$

where $B_{P}$ is the carrier loop noise bandwidth. In order to conservatively evaluate the performance degradation of the receiver under the DME interference scenario, $B_{P}$ was taken to be 20 Hz for the simulation [29], and $T_{coh2}$ was taken to represent the pre-detection integration time for carrier tracking.

**Figure 7:**
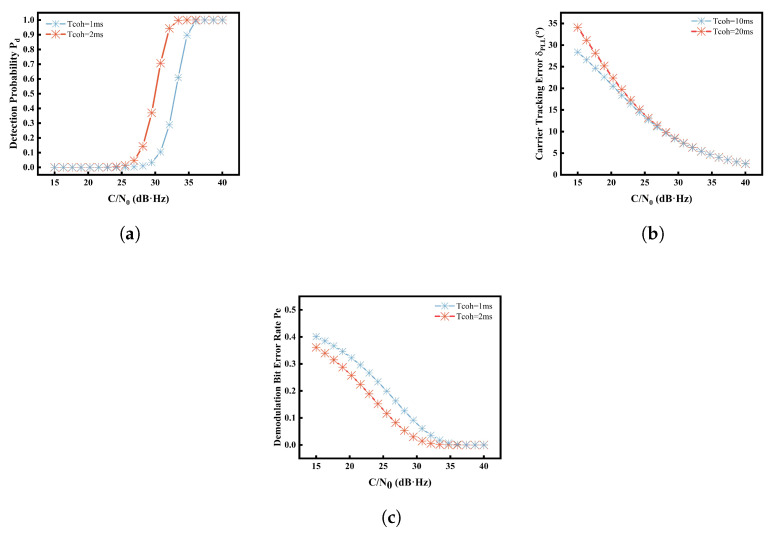
The impact of $C/N_{0}$ on the B2a signal detection probability Pd (**a**), carrier tracking error $\delta_{PLL}$ (**b**), and the demodulation bit error rate Pe (**c**).

The data demodulation of the signal is important for the performance, accuracy, and reliability of the whole navigation system. The demodulation bit error rate (BER) quantifies the probability of bit errors (Pe) during the demodulation of navigation signal data, and it is a key metric for assessing signal demodulation performance [5]. When the B2a signal is affected by interference from DME pulses, the equivalent noise power is assumed to be $\delta_{eq}^{2}$. The equivalent noise can be approximated as a Gaussian noise with mean 0 and a variance of $\delta_{eq}^{2}$. Assuming the receiver’s judgment threshold is 0, the Pe can be expressed as

(18) $$P_{e}=Q\left(\frac{\sqrt{C}}{\delta_{\mathrm{eq}}}\right)=Q\left(\sqrt{2T_{coh3}C/N_{0}}\right),$$

where *C* represents the signal power, and $T_{coh3}$ represents the integration duration of the carrier tracking loop. Different integration times were selected to carry out the simulations to verify the usability of the model, and the variations of Pd, $\delta_{PLL}$, and $P_{e}$ as functions of $C/N_{0}$ under different integration times are shown in Figure 7.

The results reveal a pronounced degradation in signal reception performance when the carrier-to-noise ratio drops below a certain threshold (30–35 dB·Hz). Specifically, the signal detection probability decreases significantly, while the carrier tracking error and demodulation bit error rate exhibit an increasing trend. However, the detection probability is more sensitive to changes. Notably, increasing the pre-detection integration time can partially mitigate the degradation in detection probability and demodulation error rate, particularly for the detection probability. This demonstrates that interference can significantly degrade receiver performance, but adjusting the integration time can influence the extent of this degradation.

## 4. Evaluation of B2a Signal Reception Performance

Building upon the established evaluation framework, this section quantitatively assesses B2a signal reception performance degradation under representative high-altitude flight conditions (FL400). The analysis integrates DME beacon network data (including transmit power, spatial distribution, etc.), propagation losses, and receiver characteristics to simulate the interference evaluation results at FL400.

The RFI threat to the B2a signal primarily originates from DME beacons co-located with VOR stations. According to the latest 2022 statistics from the Civil Aviation Administration of China (CAAC) on en-route radio navigation aids in the Chinese mainland, there are currently 274 operational DME ground beacons, with 212 co-located with VORs. After excluding 98 beacons that present no direct interference risk, the evaluation of DME beacon impacts on B2a signal focuses on 176 ground-based beacons operating in the 64X–126X channels that are co-located with VOR stations.

### 4.1. DME Signal Reception Power Budget

The signal power budget constitutes a critical component of the interference evaluation framework, necessitating comprehensive characterization of the complete transmission channel from DME beacon emission to receiver acquisition.

According to the “MH/T4006.3-1998 Aviation Radio Navigation Equipment Part 3: Rangefinder (DME) Technical Requirements” [30], the peak output power of DME beacons is specified as 1000 W (90 dBW). The maximum antenna gain is 9 dB (at an elevation angle of 8°), and the feed line loss $L_{line}$ is −3 dB.

The dominant factor in signal power attenuation is space loss in the propagation path, which comprises four key components: free-space path loss, lens effect loss, polarization, and rain attenuation loss. From the radar equation, the free-space path loss of the transmitted signal from DME beacon is calculated as follows:

(19) $$L_{loss}=10\log\left(\frac{\lambda^{2}}{{\left(4\pi R\right)}^{2}}\right),$$

where λ represents the wavelength of the signal emitted by the DME beacon, and *R* represents the slant distance from the DME beacon.

While DME signal propagation is fundamentally limited by line-of-sight constraints, ground station transmissions located slightly beyond the radio horizon remain detectable. This phenomenon occurs due to terrain diffraction effects at the horizon boundary, although additional losses due to this diffraction effect mount rapidly. In high-altitude analysis, in addition to calculating the path loss using Formula (19), additional losses caused by terrain diffraction and multipath effects due to terrain and distance must also be considered. When the terrain on the radio horizon just grazes the propagation path, an additional loss of 7-to-10 dB may occur [13].

The lens effect loss is the different degree of refraction of radio waves radiated from different elevation angles of the earth’s surface, resulting in signal loss. For the DME signal, when the antenna elevation angle is 0°, the lens effect loss $L_{lens}$ is as shown in Table 1.

The antenna polarization loss and rain attenuation loss for the L-band signal is generally set to 1 dB [18].

**Figure 8:**
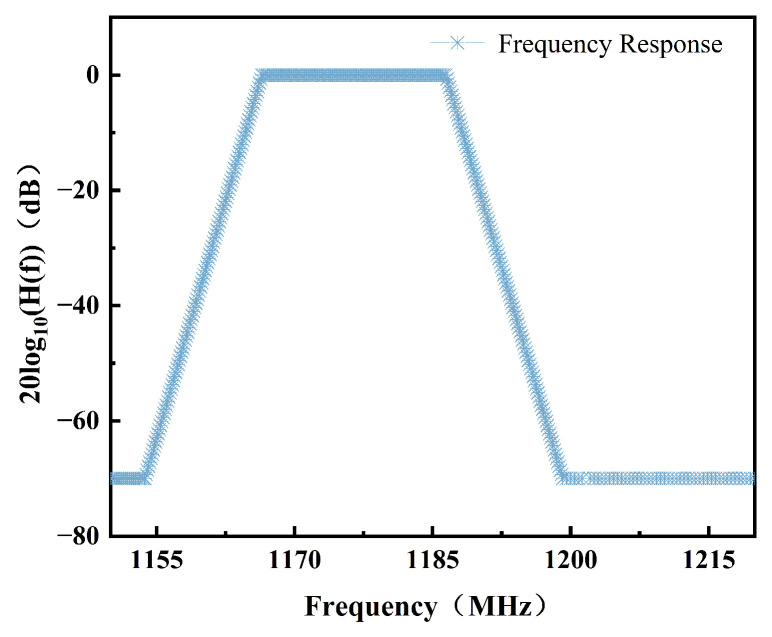
Equivalent filter transfer function response characteristics.

Within the receiver part, the receiver pre-selection filter insertion loss $L_{GNSS}$ is a key consideration. When the bandwidth is 20 MHz, the equivalent filter transfer function response characteristics of the two front-end filters and the antenna are as shown in Figure 8 [13]. The gain of the receiver antenna for the DME signals was conservatively estimated at 0 dB.

Through the above analysis, the DME signal power $P_{k}$ received by the airborne BDS receiver is

(20) $$P_{k}=89+G_{antenna}-L_{line}-L_{loss}-L_{lens}-L_{GNSS}.$$

### 4.2. Evaluation Results

A simulation evaluation platform for the B2a signal reception performance under DME interference was built based on the empirical data from the DME beacon network. The B2a signal reception performance degradation and $P_{k}$ budget were considered. The platform divides the areas from 10°-to-50° N latitude and 70°-to-140° E longitude into 1000 × 1000 grids. For each grid cell, the process begins with budgeting the received power of the DME signal, followed with using the interference evaluation model to calculate the degradation in B2a signal reception performance, and, finally, the heatmap values are then visualized.

**Figure 9:**
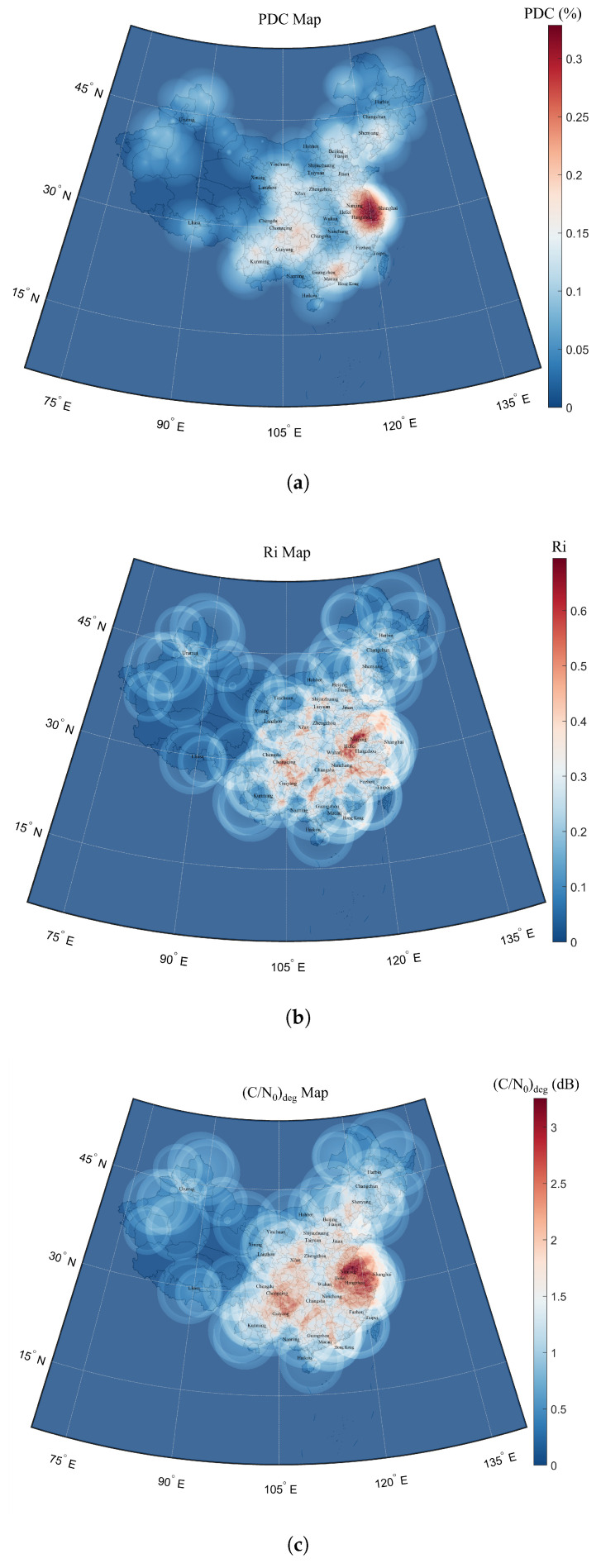
A heatmap of PDC (**a**), $R_{i}$ (**b**), and ${\left({C/N}_{0}\right)}_{\deg}$ (**c**) degradation at FL400 altitude.

Figure 9 shows the spatial distribution of effective $C/N_{0}$ degradation for the B2a signal induced by DME beacons across the Chinese mainland at an FL400 altitude, approximately 12,192 meters above standard barometric altitude. The calculations of PDC, Ri and ${\left({C/N}_{0}\right)}_{\deg}$ only consider the interference from DME signals, with $I_{0,\mathrm{WB}}=0$.

**Figure 10:**
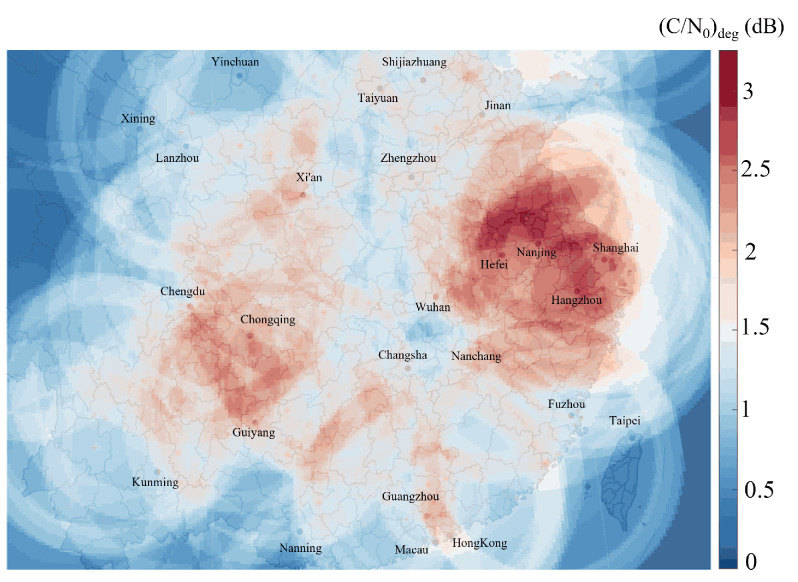
The DME interference hotspots in the Chinese mainland at FL400 altitude.

Figure 10 indicates that the most severe effective $C/N_{0}$ degradation at FL400 altitude occurs in China’s major aviation hubs, particularly the Yangtze River Delta (e.g., Shanghai, Nanjing, and Hangzhou), the Pearl River Delta (Guangzhou), and the area around Chongqing. These regions exhibit both the high air traffic density and stringent navigation precision requirements, and they are particularly susceptible to significant DME interference. The peak degradation value of 3.25 dB in ${\left({C/N}_{0}\right)}_{\deg}$ occurs at 32.9° N, 118.4° E (a location proximate to Nanjing, Jiangsu Province). In the worst-case scenario, the airborne antenna can receive signals from up to 17 DME ground beacons. Parameters for several areas experiencing severe interference are presented in Table 2.

**Figure 11:**
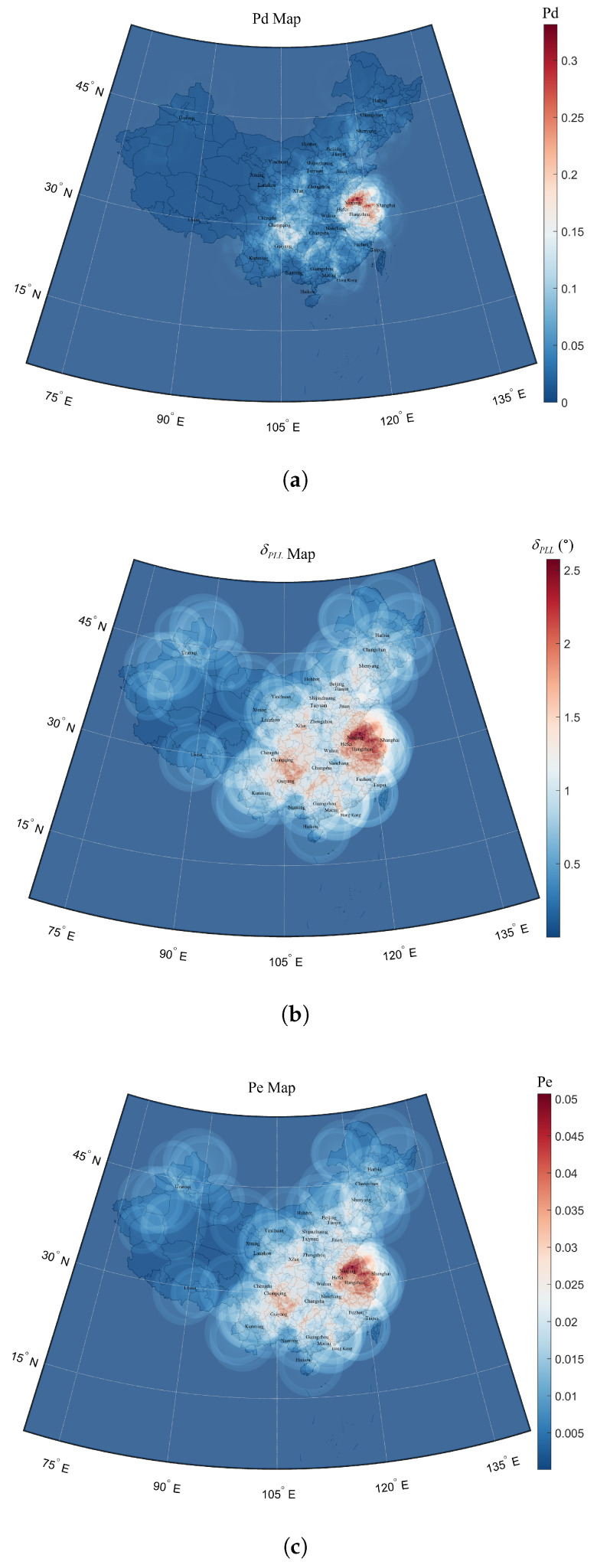
Heatmap of the B2a signal detection probability Pd (**a**), carrier tracking error $\delta_{PLL}$ (**b**), and the demodulation bit error rate Pe (**c**) degradation at FL400 altitude.

The theoretical $C/N_{0}$ at the front end of the B2a receiver is 33.898 dB·Hz without the interference of the DME [17]. Taking into account the ${\left({C/N}_{0}\right)}_{\deg}$ caused by DME, the remaining $C/N_{0}$ is calculated and can be used for further evaluation of B2a signal reception performance degradation. The pre-detection integration times for the acquisition and tracking phases were set to $T_{coh1}=2$ ms and $T_{coh2}=10$ ms respectively, while the carrier tracking loop integration time for demodulation was set to $T_{coh3}=1$ ms to specify the worst scenario. The results of B2a signal reception performance degradation are shown in Figure 11.

The spatial distribution of the B2a signal reception performance degradation at FL400 altitude strongly correlated with regions exhibiting significant effective $C/N_{0}$ degradation. These interference hotspots predominantly coincide with China’s developed regions and busy air traffic zones, where DME ground beacon density reaches high levels. The most severely interfered region was located at 32.9° N, 117.4° E (near Nanjing, Jiangsu Province), and it experienced a 33% decrease in signal detection probability, a 2.57° increase in carrier tracking error, and a 5.1% increase in demodulation bit error rate. Parameters for other severely interfered areas are presented in Table 3.

## 5. Conclusions

Building upon receiver signal processing principles, this study establishes a comprehensive interference evaluation model capable of quantitatively evaluating B2a signal performance degradation in DME interference scenarios. The model extends the conventional RTCA assessment framework and explicitly formulates the computational method for cumulative interference effects in multi-DME scenarios. The evaluation systematically analyses the interference effects and hotspot distributions through four key metrics: $C/N_{0}$, signal detection probability, carrier tracking accuracy, and demodulation bit error rate (BER). The analysis utilizes empirical data from the DME beacon network in the Chinese mainland combined with an interference budget model.

The evaluation results reveal substantial degradation in the B2a signal reception performance within regions characterized by dense DME beacon deployments. Moreover, interference hotspots show significant overlap with regions that have high air traffic density. Under the worst-case interference conditions, the effective $C/N_{0}$ of B2a signal decreases by 3.25 dB, leading to a 33% reduction in signal detection probability, a 2.57° increase in carrier tracking error, and a 5.1% increase in demodulation bit error rate. The degradation in navigation signal reception performance adversely impacts the accuracy, availability, continuity, and integrity of the navigation system, jeopardizing the flight safety of civil aviation aircraft. Furthermore, the hotspot mapping evaluation method proposed in this study is also applicable to assessing the interference effects of other L-band signals (e.g., TACAN, JTIDS/MIDS, and ADS-B) on the BeiDou navigation system.

## Figures and Tables

**Table 1:** The lens effect loss of the DME signal.

Distance (km)	$L_{\mathrm{lens}}\left(\mathrm{dB}\right)$
50	0.05
100	0.1
500	0.18
300	0.3
350	0.5

**Table 2:** The PDC, $R_{i}$, and ${\left(C/N_{0}\right)}_{\deg}$ parameters at FL400 altitude in major areas.

Region	Latitude and Longitude	PDC	Ri	${\left({\mathrm{CN}}_{0}\right)}_{\deg}\left(\mathrm{dB}\right)$
Beijing	39.9° E, 116.3° N	0.14	0.23	1.52
Hefei	31.9° E, 117.3° N	0.23	0.39	2.55
Nanjing	32.1° E, 118.1° N	0.26	0.35	2.64
Shanghai	31.4° E, 121.8° N	0.25	0.38	2.65
Chengdu	30.6° E, 104.1° N	0.17	0.28	1.88
Chongqing	29.9° E, 106.8° N	0.18	0.35	2.17
Guangzhou	23.3° E, 113.4° N	0.22	0.28	2.12
Maximum ${\left({C/N}_{0}\right)}_{\deg}$	32.9° E, 118.4° N	0.23	0.63	3.25

**Table 3:** $P_{d}$, $\delta_{PLL}$, and $P_{e}$ parameters at the FL400 altitude in major areas.

Region	Latitude and Longitude	$P_{d}\left(\%\right)$	$\delta_{\mathrm{PLL}}{(}^{{}^{\circ}})$	$P_{e}\left(\%\right)$
Beijing	39.9° E, 116.3° N	3	1.06	1.8
Hefei	31.9° E, 117.3° N	17	1.91	3.6
Nanjing	32.1° E, 118.1° N	19	1.99	3.8
Shanghai	31.4° E, 121.8° N	19	2.01	3.8
Chengdu	30.6° E, 104.1° N	7	1.35	2.4
Chongqing	29.9° E, 106.8° N	10	1.59	2.9
Guangzhou	23.3° E, 113.4° N	10	1.55	2.8
Maximum ${\left({C/N}_{0}\right)}_{\deg}$	32.9° E, 118.4° N	33	2.57	5.1

## Data Availability

The data presented in this study are available on request from the corresponding authors.
